# Polypseudophakia: from “Piggyback” to supplementary sulcus-fixated IOLs

**DOI:** 10.1007/s00417-024-06618-3

**Published:** 2024-09-11

**Authors:** Ramin Khoramnia, Guenal Kahraman, Michael Amon, Grzegorz Łabuz, Isabella D. Baur, Gerd U. Auffarth

**Affiliations:** 1 Department of Ophthalmology, Heidelberg University Hospital, Im Neuenheimer Feld 400, 69120 Heidelberg, Germany; 2 Academic Teaching Hospital of St John, Johannes-Von-Gott-Platz 1, 1020 Vienna, Austria; 3 Department of Ophthalmology, Sigmund Freud Private University Vienna, Faculty of Medicine, Campus Prater, Freudplatz 1+3, A-1020 Vienna, Austria

**Keywords:** Supplementary IOLs, Piggyback, Pseudophakia, Sulcus-fixated, DUET

## Abstract

**Abstract:**

Polypseudophakia, the concept of using a second intraocular lens (IOL) to supplement an IOL that has already been placed in the capsular bag, was first used as a corrective measure where the power requirement was higher than that of available single IOLs. Subsequently, the technique was modified to compensate for post-operative residual refractive errors. In these early cases, an IOL designed for the capsular bag would be implanted in the sulcus.  Although these approaches were less than ideal, alternative means of correcting residual refractive errors were not without their limitations: IOL exchange can be traumatic to the eye and is not easily carried out once fibrosis has occurred, while corneal refractive surgical techniques are not suitable for all patients. Piggyback implantation was the term first coined to describe the use of two IOLs, placed together in the capsular bag. The term was later extended to include the procedure where an IOL designed for the capsular bag was placed in the sulcus. Unfortunately, the term piggyback has persisted even though these two approaches have been largely discredited. Intraocular lenses are now available which have been specifically designed for placement in the ciliary sulcus. As these newer IOLs avoid the many unacceptable complications brought about by both types of earlier piggyback implantation, it is time to employ a new terminology, such as supplementary IOL or secondary enhancement to distinguish between the placement of an unsuitable capsular bag IOL in the sulcus and the implantation of an IOL specifically designed for ciliary sulcus implantation. In addition to minimising possible complications, supplementary IOLs designed for the sulcus have expanded the options available to the ophthalmic surgeon. With these new IOLs it is possible to correct presbyopia and residual astigmatism, and to provide temporary correction of refractive errors in growing, or unstable, eyes. This article aims to review the literature available on supplementary IOL implantation in the ciliary sulcus and to summarise the evidence for the efficacy and safety of this intervention.

**Key messages:**

***What is known***

- Polypseudophakia has been used for over 30 years to correct hyperopia or residual refractive error, but early techniques were associated with significant complications.

***What is new***

- The development of specially designed sulcus-fixated supplementary IOLs significantly reduces the risks associated with these procedures, and has also opened up new opportunities in patient care.
- The reversibility of the procedure allows patients to experience multifocality, and to provide temporary and adjustable correction in unstable or growing eyes.
- The terms “secondary enhancement” or “DUET” to describe supplementary IOL implantation are preferential to “piggyback”.

## Historical use of polypseudophakia to achieve optical power

In the ‘two-in-the-bag’ technique, first reported in 1993, an additional IOL is inserted in the capsular bag over the existing lens implant (hence the term “piggyback”) to adjust the optical power.[1] Primary implantation of a second IOL was initially used for extreme hyperopia when a single high power IOL was out of the range of manufacturers’ power inventories and those available were inadequate to provide sufficient power.[1, 2] This became unnecessary once manufacturers routinely developed IOLs with higher dioptric power.

In 1999 the first case report emerged of a multifocal IOL being implanted two-in-the-bag with an aspheric IOL (both three-piece silicone) to simultaneously correct hyperopia and improve near vision.[3] This was followed in 2001 by the same technique used in five patients.[4]

This procedure was used throughout the 1990s but it was discovered that placing two lenses in the bag allowed an avenue for lens epithelial cells to proliferate, resulting in visually significant interlenticular opacification (ILO).[5] It is characterised by opacification in the interlenticular space, caused by Elschnig pearls or membrane formation, resulting in a loss of corrected visual acuity.[6] As ILO is a late complication, the actual incidence is probably higher than that reported.[7]

One of the early reports of ILO took place two years after surgery in 70 eyes, in a study where two IOLs had been implanted, some two-in-the-bag, and some in the bag and the sulcus. Post-operative opacification was found in 23 eyes (32.8%), but only in cases where both IOLs had been inserted into the capsular bag.[5] The interlenticular membrane was easily stripped from between polymethylmethacrylate (PMMA) lenses (two cases) but some acrylic IOLs were tightly fused and could not be separated.[5]

The exact cause of ILO is unknown, but it has been postulated that bioadhesion of the anterior lens to the anterior capsule and the posterior lens to the posterior capsule may prevent cell migration from the equatorial bow to the posterior capsule. This migration may instead be directed toward the interlenticular space, resulting in ILO.[5] Different types of opacification have been reported, with the most common being the formation of Elschnig pearls, thought to arise from retained proliferative lens epithelial cells. In some histopathological examinations following IOL explantation, epithelial cells were mixed with cortical lens material.[5]

There also appears to be a difference in the incidence of ILO related to IOL material: incidence was found to be higher when acrylic IOLs were used compared with PMMA IOLs.[6]

Although the mechanism is not entirely clear, hyperopic shift can take place when two IOLs are placed in the capsular bag, which may arise from contact between the two optics leading to deformation or flattening, resulting in a lower refractive power.[5, 6] Shugar and Schwarz (1999) reported clinically significant hyperopic shift that emerged between 1 and 2 years after implantation of two IOLs in the capsular bag.[7] All eyes had visible proliferating Elschnig pearls in the peripheral interface between the IOL optics.[7] This phenomenon has not been reported where the second IOL is placed in the ciliary sulcus.

Despite these drawbacks, and the existence of better alternatives, it is surprising that this technique was still being used as recently as 2022.[8]

## Options for correction of refractive surprise

### Causes of refractive surprise

Since the development in the 1980s of optical biometry using PCI (partial coherence interferometry),[9] and following this, the emergence of SS-OCT (swept-source optical coherence tomography)[10] refractive outcomes have improved considerably with a steadily increasing percentage of patients achieving target refraction. Between 1992 and 2006, around 72 to 87% of patients deviated from target refraction by ±1.0 D, increasing to over 90% in the following ten years.[11] At the same time, between 1996 and 2005, 45 to 58% of patients were within ±0.5 D, increasing to over 61% in the following ten years.[11]

Despite these successes, refractive surprises are still not uncommon. The requirement for a secondary procedure to reduce residual refractive error after refractive lens exchange and multifocal IOL implantation has decreased in the last decade but is still in the region of 3 to 10%.[11]

There are numerous steps involved in cataract surgery any one of which can contribute to refractive surprise.

In the pre-operative stages, biometric data, such as axial length, corneal curvature (refractive power), anterior chamber depth (ACD), lens thickness, white-to-white, can be included to estimate the refractive power of the IOL to be implanted. Small errors in the measurement of biometric data can result in clinically significant refraction errors. Accurate estimation is critical as an error of 0.1 mm in axial length can equate to a post-operative refractive error of around 0.27 D,[12] while an error of 1 mm in ACD can result in an error of 1.44 D.[13] There are limitations to the available IOL power calculation formulae, which tend to work best in eyes with a normal axial length.[14] Aristodemou et al (2011) evaluated the most commonly used IOL formulae and found that only 75% of eyes were within ±0.5 D of target refraction, despite using the optimised A constant.[15]

Mis-estimation of the post-operative IOL effective lens position, broad tolerances in the labelling of IOLs, previous eye surgery, and pre-existing corneal astigmatism (at least 1.0 D in around one-third of cataract surgery patients) all contribute to post-operative refractive error.[14]

Surgical factors such as the size and position of the capsulorhexis are important as is the avoidance of surgically induced astigmatism.[11] Following surgery, rotation, decentration and tilt will affect post-operative outcomes, as will capsular fibrosis and contraction leading to anterior movement.[16]

### IOL exchange for correction of refractive error

In IOL exchange, the primary IOL is removed and replaced with one of the correct power. It is best performed early, before adhesions have formed and used to be preferred over other methods for the correction of large errors.[17] Late removal can lead to complications such as posterior capsular rupture and zonular damage which in turn can lead to cyclodialysis, retinal tears and macular oedema.[18] Damage to the zonulo-capsular complex may prevent fixation of another IOL, possibly requiring additional surgery for a scleral- or iris-fixated or angle-supported IOL [17, 19, 20]

Another drawback with IOL exchange is that it is difficult to estimate the level of correction required without knowing the true cause of the original error. If the refractive issue was due to incorrect IOL power selection, the exact cause must be identified, and the magnitude of the error calculated, if lens exchange is to be performed.[18]

### Corneal refractive surgery to correct residual error

Corneal refractive surgery, (or laser vision correction surgery) is a safe, effective, predictable and non-invasive method to correct residual refractive error following cataract surgery,[21] and is particularly applicable when there is stable refractive error, and normal corneal topography.[22] It can be particularly successful when the residual refractive error is myopia.[23, 24] Intrastromal arcuate keratotomy is also reported to achieve good results.[25]

However, laser-based techniques are not as effective as lens-based procedures in the correction of large refractive errors,[24] and there are other disadvantages to this technique. Specifically, laser ablation is not viable in eyes with thin corneas, abnormal corneal topography, corneal scarring or degeneration, or pre-existing HOAs (higher order aberrations).[24] In fact it has been reported that it may induce HOAs[26] and, in rare cases, secondary corneal ectasia.[27] It may not be suitable in an older population with a higher prevalence of dry eye.[28, 29] Other concerns are a higher cost and a lack of reversibility.

#### Secondary implantation for correction of refractive error

The use of two IOLs in the eye (polypseudophakia) was initially used to achieve optical power by placing two IOLs in the capsular bag. The concept of using two IOLs rapidly extended to become a technique for correction of refractive surprise, where the second IOL is placed in the sulcus rather than the capsular bag. Although these two techniques are very different, it is unfortunate that the term “piggyback” was also extended to, and came to be associated with, this procedure as well.

Secondary sulcus implantation was seen to be less traumatic than IOL exchange, particularly if the original procedure had been performed some time before, carried a lower risk of complications and produced more predictable refractive results. The capsular bag is preserved and the additional IOL power can be easily calculated based on refraction and vergence calculation, without the need to know the power of the first IOL, or the axial length of the eye.[18] It is effective in reducing high degrees of spherical error, and is reversible.[14, 30]

In one report, compared to IOL exchange, implantation of a capsular bag IOL in the sulcus resulted in 15% more eyes achieving uncorrected visual acuity (UCVA) of 20/20 or better.[18] Ninety-two per cent of eyes that underwent implantation of the additional lens achieved a spherical equivalent refraction within 0.5 D of the intended correction, compared to only 82% of eyes that underwent IOL exchange.[18] Note that the secondary IOL in this case, the hydrophobic acrylic AcrySof MA60MA, was not designed for sulcus implantation.

Compared to laser-based procedures, the implantation of a secondary IOL does not alter the anterior corneal surface or significantly change corneal refractive power,[14] which may provide more precise outcomes,[18] and may be a suitable method for patients with dry eye disease, thin corneas, or keratoconus who are not suitable for laser vision correction.[31]

However, in early reports, IOLs designed solely for use in the capsular bag, such as the AcrySof MA60MA, SA60AT and MA60BM, Storz P359UV, Staar AQ-5010V, were often inappropriately implanted in the ciliary sulcus.[16] There are many reports in the literature of the complications caused by the implantation of a standard capsular bag IOL in the sulcus.[32–35] These include: pigment dispersion,[36–38] iris transillumination defects, dysphotopsia, glaucoma,[39] intraocular haemorrhage, cystoid macular oedema, hyperopic defocus and pupil capture.[35, 40, 41]

Following a survey of committee members in 2009, the Cataract Clinical Committee of the American Society for Cataract and Refractive Surgery concluded that: “IOLs designed solely for the capsular bag should not be placed in the ciliary sulcus.[35] The haptics are generally too large, and can contact the posterior iris. They are also planar and do not vault the optic posteriorly from the iris. Finally, most are too small (13.0 mm) which can lead to decentration in a larger eye.”[35]

## Sulcus-fixated supplementary IOLs

A welcome development in recent years has been that of supplementary IOLs which have been specifically designed to avoid the complications associated with implantation of a capsular bag IOL in the ciliary sulcus.

The critical features of these IOLs which make them suitable for this application are: hydrophilic acrylic material to optimise uveal biocompatibility, a large optical diameter to avoid IOL capture during pupil dilation, a rounded optic edge to avoid visual disturbances, angulated haptics to reduce the risk of contact with the posterior surface of the iris, a concave posterior optic to avoid the risk of contact between the primary and secondary IOL, and a rounded haptic edge to minimise irritation of the iris, pigmentary dispersion, and secondary pigmentary glaucoma. Implantation of an appropriately designed supplementary IOL in the ciliary sulcus maintains effective distance between the two IOLs, minimising the risk of formation of ILO.[31]

Compared to IOL exchange which must be carried out relatively quickly after the initial procedure, implantation of a sulcus-fixated supplementary IOL, can be carried out years after the primary procedure.[42] As with the implantation of any IOL in the sulcus, power calculation depends solely on the patient’s existing subjective refractive status and not on the pre-operative biometry measurements.[16] As a result, power calculations are likely to be more accurate than for IOL exchange.[43]

The term “DUET” procedure has been suggested by Michael Amon to describe the procedure where a sulcus-designed supplementary IOL is implanted in the ciliary sulcus and a primary IOL is implanted in the capsular bag.[44] The implantations can be carried out in the same procedure, particularly for correction of high myopia or hyperopia, for presbyopia or astigmatism, or sequentially if post-surgical enhancement is needed.

### Extending the applications for supplementary IOLs

The development of appropriately designed sulcus-fixated IOLs has led to an expansion of their uses beyond the correction of residual refractive error after cataract surgery. Note that some of the following applications may be off-label for specific sulcus-fixated IOLs and have been explored by individual ophthalmologists seeking solutions for particular patient problems.

**As a primary procedure (DUET)**: for eyes with extreme refractive error, refractive power can be split between the two IOLs.[22] Any residual refractive error within the available power range of the sulcus-designed supplementary IOL can be treated by exchanging the supplementary IOL.

**Eyes with silicone oil in situ**: pars planar vitrectomy with silicone oil filling is used to stabilise the retina in complicated retinal conditions. When used as a tamponade agent, silicone oil changes the refractive status of the eye. Once the tamponade is no longer required, and the silicone oil is removed, there is likely to be a refractive shift. Implantation of a supplementary IOL can reduce the problems caused by anisometropia, allow for better UCVA, and can be easily exchanged once the oil has been removed.[22]

**Keratoconus:** eyes with keratoconus or undergoing keratoplasty often have high astigmatism but may need future procedures in response to changes in astigmatism. A spherical IOL can be implanted in the capsular bag, and a supplementary toric IOL in the sulcus to correct the initial astigmatism. The supplementary IOL can be exchanged if necessary following any repeat procedures.[22]

**Presbyopia correction**: a monofocal IOL targeted on emmetropia is implanted in the bag and a plano multifocal IOL in the sulcus to correct for presbyopia.[22] In some patients, it can be difficult to predict their tolerance to halo, glare and reduced contrast sensitivity.[45] If the patient cannot tolerate the multifocal optic, the supplementary IOL can be removed and the patient will still remain corrected for spectacle-independent distance vision.[22] This process provides an opportunity for patients to experience multifocality – or trifocality depending on the IOL – while allowing safe and easy removal of the sulcus-based IOL if side effects prove intolerable.[45] Some patients cannot adjust to the multiple images, photic phenomena or reduced contrast sensitivity (CS) of the multifocal optic.

**Paediatric applications**: IOL implantation in an infant, for example, for aphakia or following trauma, is common, with the aim usually being to achieve emmetropia.[22] But as the child grows, the eye will become increasingly myopic. To counter this, the power of the IOL in the bag is calculated to target emmetropia once the eye is fully grown, while a supplementary IOL can compensate for the intermediate period while the eye is growing.[22] As the eye changes, the supplementary IOL can be removed or exchanged.

**Other eye pathologies:** patients with cataract at a young age may develop ocular pathologies such as macular degeneration, glaucoma, or retinal detachment, leading to loss of function. Implantation of a multifocal or trifocal IOL in the bag has disadvantages as the condition progresses, but a supplementary IOL can be safely removed, even decades after surgery.[45]

## Currently available supplementary sulcus fixated IOLs

Supplementary IOLs are available from the following manufacturers: Rayner (Sulcoflex range), 1stQ / Medicontur (AddOn range), Cristalens Industrie (Reverso), and Morcher (XtraFocus pinhole supplementary IOL). Details are summarised in Tables 1 and 2.

**Table 1:** Currently available sulcus-fixated supplementary IOLs

IOL	Structure	Material	Optic diameter	Optic features	Overall diameter	Haptics	Angulation
Sulcoflex (Rayner)	One-piece	Hydrophilic + UV filter (Rayacryl)	6.5 mm	Round edges. Concave posterior	14.0 mm	Undulating, rounded edges	10^o^
AddOn (1stQ)	One-piece	Hydrophilic + UV filter	6.0 mm	Round edges. Convex-concave	13.0 mm	Four flexible closed loops, 0.3 mm thick	0
Reverso (Cristalens)	One-piece	Hydrophilic (Benz 25)	6.5 mm	Round edges. Convex-concave	13.8 mm	Open C loop	10^o^
XtraFocus (Morcher)	One-piece	Black hydrophobic acrylic	1.3 mm, occluded Sect. 6 mm	Concave-convex	14.0 mm	Undulating, rounded, polished edges	14^o^

Sulcoflex Technical Information sheets available from Rayner Intraocular Lenses Ltd, 10 Dominion Way, Worthing, West Sussex BN14 8AQ, UK. 1stQ IOL Portfolio data sheets (2024) from www.1stq.de/download. Cristalens Technical Specification available from Cristalens Industrie, 4 Rue Louis de Broglie, 22,300 Lannion, France. XtraFocus technical brochure available from Morcher GmbH, Kapuzinerweg 12, 70,374 Stuttgart, Germany

**Table 2:** Power ranges for currently available sulcus-fixated IOLs

	**IOL**	**Range**	**Increment**
Rayner Sulcoflex	Sulcoflex® Aspheric 700L (formerly 653L)	-5.0 to + 5.0 D (standard range)	0.50 D
	Sulcoflex® Toric 710 T (formerly 653 T)	Standard SE: -3.0 to + 3.0 D Cylinders: + 1.0 D, + 2.0 D, + 3.0 D Made to order: SE: -7.0 to + 7.0 D Cylinders: + 1.0 to 6.0 D	0.50 D
	Sulcoflex® Multifocal 653F (bifocal refractive) with an addition of + 3.50 D. Discontinued in most markets	Sphere from -3.0 to + 3.0 D	0.50 D
	Sulcoflex® Trifocal 703F (trifocal diffractive) with a near addition of + 3.50 D, intermediate addition of + 1.75 D	-3.00 to + 3.00 D -1.00 to + 1.00 D	0.50 D 0.25 D
1stQ AddOn®	Spherical	-0.5 to + 0.5 D -10.0 to -0.5 D + 0.5 to + 10.0 D	0.50 D 0.25 D 0.25 D
	Toric	-10.0 to + 10.0 D sphere + 1.0 to + 11.0 D cylinder	
	Progressive (multifocal, EDOF, trifocal), with different additions + 2.25 D; + 3.0 D to mix and match	-5.0 to + 5.0 D	
	Progressive trifocal-toric with a near addition of + 3.0 D	-3.0 to + 3.0 D sphere + 1.0 to + 4.5 D cylinder	
	SML (Scharioth Macula Lens) addition for ultra-near vision: ARMD	On request	
Cristalens Reverso®	Reverso® Monofocal	-6.0 to + 6.0 D	0.50 D
	Reverso® Multifocal Available with different additions of + 1.50 D, + 2.00 D, + 2.50 D, + 3.00 D, + 3.50 D to mix and match	-3.0 to + 3.0 D	0.50 D

ARMD: age-related macular degeneration; EDO: extended depth of focus

Sulcoflex Technical Information sheets available from Rayner Intraocular Lenses Ltd, 10 Dominion Way, Worthing, West Sussex BN14 8AQ, UK. 1stQ IOL Portfolio data sheets (2024) from www.1stq.de/download. Cristalens Technical Specification available from Cristalens Industrie, 4 Rue Louis de Broglie, 22,300 Lannion, France. XtraFocus technical brochure available from Morcher GmbH, Kapuzinerweg 12, 70,374 Stuttgart, Germany

In addition, HumanOptics used to market the Aspira range of supplementary IOLs. These were three-piece silicone IOLs, available as aspheric, toric and multifocal models and achieved good clinical results as reported in a number of studies.[46–53] This range has now been discontinued and is no longer available.

The Morcher XtraFocus is an opaque disc with a pinhole 1.3mm opening, usually applied for the correction of irregular astigmatism due to keratoconus, pellucid marginal degeneration, perforating corneal trauma, other corneal irregularities or following radial keratotomy or PK (penetrating keratoplasty). The small aperture blocks most peripheral aberrated rays and the pinhole effect can extend the depth of focus which may be helpful for the correction of presbyopia.[54, 55] It is included here for completeness.

A low level of complications has been reported for these four IOLs. These include dysphotopsia, transiently raised postoperative intraoperative pressure, and mild cases of pigment dispersion that did not require treatment. No cases of iris chafing, or ILO were reported.

See Table 3 for a summary of all the studies referred to in this section.

**Table 3:** Published studies of sulcus-fixated IOLs

Study / type of study / IOL	N, mean age / follow-up	SE	Predictability	Visual acuity	Multifocality / CS	Astigmatism	Complications including dysphotopsia	Stability
**Sulcoflex range**								
Baur et al. (2022)[45] Retrospective chart review Sulcoflex Trifocal 703F	48 eyes, 25 patients 3 months		Target -012 ± 0.11 D Achieved -0.14 ± 0.31 D Difference not statistically significant	**Distance:** Mean pre-operative UDVA: 0.71 ± 0.43 logMAR Mean post-operative: 0.04 ± 0.10 logMAR CDVA: Mean pre-operative: 0.12 ± 0.16 logMAR Mean post-operative: 0.01 ± 0.09 logMAR	**Near:** Mean pre-operative UNVA: 0.86 ± 0.27 logMAR Mean post-operative: 0.06 ± 0.08 logMAR UIVA: Pre-operative not reported Mean post-operative: 0.00 ± 0.10 logMAR **Defocus curve:** Monocular: VA of at least 0.2 logMAR from + 0.75 to -3.5 D Binocular: VA of at least 0,2 logMAR from + 1.0 to -3.75 D		Suture fixation required in one eye All patients experienced halos, starburst was most common (10/14) Eight patients reported glare	
Falzon and Stewart (2012)[16] Retrospective chart review Sulcoflex 653L × 3 653 T × 12	15 eyes, 13 patients Mean: 63 years (52 – 81) Mean 20 months (14 – 30)	Mean pre-operative: -0.54 ± 1.11 D Mean post-operative: -0.15 ± 0.28 D	All within 1.0 D of attempted correction, 93% within 0.05 D	**Distance:** Mean pre-operative UDVA: 0.44 logMAR Post-operative: all eyes 0.20 logMAR or better		Mean pre-operative error: -1.45 ± 0.98 D Mean post-operative: -0.50 ± 0.57 D	None significant One eye had increased IOP at 1 month. Three eyes had AC flare at 1 month: resolved with treatment	All eyes well centred, no decentration or tilt
Ferreira et al. (2015)[31] Prospective case series Sulcoflex 653 T	10 eyes, 10 patients Mean: 56.42 years (45 – 65) Mean 6.99 months (6 – 18)	Mean pre-operative: -1.77 ± 2.64 D Mean post-operative: -0.30 ± 0.56 D (p = 0.001) Mean spherical power implanted: -2.28 ± 2.80 D Mean cylinder: + 3.14 ± 1.15 D	Spherical refraction within ± 0.50 D of target in 5 eyes and within ± 1.00 D in 9 Refractive cylinder within ± 0.50 D in 5 eyes and within ± 1.00 D in 8	**Distance:** Mean post-operative: 0.10 ± 0.12 logMAR (p = 0.004) Mean post-operative CDVA: 0.07 ± 0.12 logMAR (p = 0.021) Nine eyes gained lines of CDVA		Mean pre-operative refractive astigmatism was -2.96 ± 0.84 D		Mean axis misalignment at 6 months 3.0 ± 2.45^o^
Kahraman and Amon (2010)[56] Prospective, non-randomised Sulcoflex 653L	12 eyes, 10 patients Median: 53.58 years Mean 12 months (6 – 17)	Mean pre-operative: -1.25D (-2.00 to + 4.00 D) Mean post-operative: -0.25D (-0.50 to + 0.25 D)		**Distance:** Mean post-operative UDVA: 0.9 ± 0.1 Snellen			No signs of pigment dispersion, iris bulging, foreign-body giant cell formation, or ILO	Slit-lamp: Decentration in one eye of < 0.5 mm. No rotation or tilt. Stable distance between primary IOL
Kahraman et al. (2021)[57] Prospective, non-randomised Sulcoflex Trifocal 703F	40 eyes, 20 patients 69.58 years (56 – 80) 1 to 6 months	SE not reported All implanted IOLs were 0.0 D, near addition + 3.50 D intermediate addition + 1.75 D		**Distance:** Mean post-operative UDVA: -0.07 ± 0.06 logMAR Mean pre-operative CDVA: 0.20 ± 0.06 logMAR Mean post-operative: -0.08 ± 0.07 logMAR	**Intermediate:** Mean post-operative (6 months) UIVA: -0.03 ± 0.17 logMAR **Near:** UNVA: 0.09 ± 0.08 logMAR			No rotation or tilt
Khan and Muhtaseb (2011)[43] Case series Multifocal × 4 Toric × 1	5 eyes, 4 patients 6 months			**Distance:** > 0.1 logMAR in all cases	**Near:** UNVA of N6 in all multifocal eyes		No iris chaff, or ILO	
Levinger et al. (2020)[58] Retrospective cohort Sulcoflex 653L × 13 Sulcoflex 653 T × 2	15 eyes 70 years (51 – 86) Mean 14 months (3 – 18)	Mean pre-operative: -0.22 ± 5.95 D Mean post-operative: -1.59 ± 1.45 D	Mean deviation -1.38 D (-0.50 to -3.0)	**Distance:** Mean pre-operative UDVA: 0.88 logMAR Improved in all patients, with eight reaching emmetropia				All aspheric IOLs remained centred, no tilt or rotation One toric IOL had slight decentration
McIntyre et al. (2012)[59] Retrospective, cadaver eyes Sulcoflex 653L	11 of 16 eyes 77.16 years (69—83) n/a							Ultrasound analysis: Good centration, minimal or no tilt Clearance: 232 – 779 µm
McLintock et al. (2019)[60]** Retrospective chart review Sulcoflex 653 T	51 eyes		64% were within 0.5 D of target SE 53% were within 0.5 D of target cylinder	**Distance:** Mean pre-operative UDVA: 20/86 Mean post-operative: 20.43 (p = 0.002)				Mean rotation on day one was 8.23^o^
Prager et al. (2017)[61] Retrospective case series Not specified	48 eyes, 43 patients Mean 25 months (12 – 84)							Slit-lamp photography: Mean decentration: 0.23 ± 0.02 mm (limbus), and 0.22 ± 0.02 mm (pupil)
Schrecker et al. (2014)[51] Prospective, randomised Sulcoflex 653F (35) MS 714 PB Diff (33)	68 eyes, 40 patients 3 months		Median deviation of -0.11 D (-0.32 to 0.57 D)	All eyes achieved at least 0.3 logMAR at all distances Percentage of eyes achieving 20/25 higher in the MS group (88%) than the Sulcoflex group (66%)	Mean CS values within normal range under photopic conditions with and without glare, better in the MS Diff group	At 3 months, mean 0.50 D (0.10 to 1.30 D)	Mild pigment dispersion, requiring no treatment, seen in 6 eyes Visual disturbances at night in 81% (Sulcoflex) and 25% (MS Diff), mostly mild or moderate and not causing severe disturbance	No noticeable decentration or tilt
Venter et al. (2014)[62] Retrospective review Sulcoflex 653L	80 eyes, 64 patients 59.8 years (41 to 81) 12 months	Mean pre-operative: + 0.58 ± 1.15 D Mean post-operative: -0.14 ± 0.28 D Power range used: -2.50 to + 4.50 D	93.8% within 0.5 D of emmetropia, and 98.8% within 1.0 D	**Distance:** Mean pre-operative UDVA: 0.28 ± 0.16 logMAR Mean post-operative: 0.01 ± 0.10 logMAR	**Near:** Mean pre-operative UNVA: 0.43 ± 0.28 logMAR mean post-operative: 0.19 ± 0.15 logMAR		No serious complications Three cases of iritis managed with steroids Raised IOP in 7 patients, resolved	
**AddOn range**								
Albayrak et al. (2021)[63] Prospective non-comparative Trifocal 677MY	28 eyes, 18 patients 6 months		89% within 1.0 D of target emmetropia	**Distance:** Mean pre-operative UDVA: 0.21 ± 0.34 logMAR Mean post-operative UDVA: 0.05 ± 0.08 logMAR Mean pre-operative CDVA: 0.14 ± 0.22 logMAR Mean post-operative CDVA: 0.01 ± 0.03 logMAR	**Intermediate:** Mean pre-operative UIVA: 0.40 ± 0.12 logMAR Mean post-operative UIVA: 0.06 ± 0.020 logMAR, (*p* = 0.0204) **Near:** Mean pre-operative UNVA: 0.50 ± 0.23 logMAR Mean post-operative UNVA: 0.02 ± 0.05 logMAR, (*p* = 0.0104) CS within normal range but reduced compared to pre-operative values		None, such as iris chafing, iris capture, interlenticular opacification No dysphotopias	No tilt, decentration or other misalignment
Gundersen et al. (2017)[64] Retrospective chart review Aspheric 36 Toric 10	46 eyes Approx. 59 (actual mean not given) Up to 3 months	Mean post-operative SE refraction was − 0.25 D Absolute reduction in SE was 0.36 ± 0.30 (p < 0.01)	Target was emmetropia 76% of eyes within 0.50 D of target; 57% within 0.25 D	**Distance:** Significant improvement of 2 lines (p < 0.01) CDVA: all eyes had at least 0.1 logMAR		60% of eyes had ≤ 0.25 D residual refractive astigmatism Statistically significant reduction in eyes fitted with Toric IOL (p < 0.01)		
Gundersen et al. (2020)[65] Non-interventional diagnostic Toric IOL	18 eyes Minimum 1 month (43 days to 4.5 years)	Mean pre-operative SE: -0.18 ± 0.88 D Mean post-operative: -0.15 ± 0.44 D (no significant change) Cylinder: Mean pre-operative: -1.66 ± 0.93 D Mean post-operative: -0.32 ± 0.25 D Mean sphere power 0.03 ± 1.23 D 72% had cylinder power of 1.5 (7) or 2.25 (6)		**Distance:** Mean post-operative UDVA: 0.00 ± 0.03 logMAR 76% had a UDVA of plano (0.0 D) or better No significant change in CDVA		Mean residual refractive astigmatism significantly reduced from 1.66 ± 0.92 to 0.32 ± 0.25 D (p < 0.001) 89% had residual refractive astigmatism ≤ 0.50 D	No statistically significant difference in IOP	SS-OCT Mean absolute lens rotation of ≤ 5°: -0.1 ± 6.3^o^ 89% of eyes had a lens rotation of ≤ 10°
Harrisberg et al. (2023)[66] Retrospective cohort study Trifocal A4DWOM, plano	32 eyes, 18 patients Control 57 eyes (not reported) Up to 1 year		95% within ± 0.5 D of target SE	**Distance:** UDVA: 91% within 0.2 logMAR at one year 71% within 0.3 logMAR at one year			One case of pupillary capture requiring correction Three cases of IOP > 30 mmHg not requiring intervention	All well-centred, no tilt
Palomino-Bautista et al. (2020)[42] Prospective observational Trifocal 677MY	18 eyes, 11 patients 73.1 years (61 – 83) 6 months		83.3% of eyes within ± 0.5 D of emmetropia (pre-operative: 16.7%) All eyes had SE refractions within ± 1.0 D of target refraction	**Distance:** Mean pre-operative UDVA: 0.21 ± 0.11 logMAR Mean post-operative: 0.03 ± 0.05 logMAR	**Intermediate:** Mean pre-operative UIVA not measured Mean post-operative: UIVA 0.21 ± 0.04 logMAR **Near:** Mean pre-operative UNVA: 0.51 ± 0.15 logMAR Mean post-operative: 0.12 ± 0.04 logMAR Spectacle independence achieved in all eyes at all distances		No negative effects, eg endothelial cell count, IOP, corneal or iris damage, pupillary damage, pigment dispersion	No tilt, decentration, rotation or dislocation
Reiter et al. (2017)[67] Cadaver study A45D and A45SML AddOn	12 cadaver eyes 88.6 years							Anterior segment optical coherence tomography No cases of decentration Four cases of tilt Interlenticular distance: 0.34 to 1.24 mm
**Reverso IOL**								
Cassagne et al. (2018)[68] Prospective case series	54 eyes, 27 patients 69 years (53 – 84) Up to 1 year	At 1 year mean manifest refraction SE was -0.02 to 0.61 D	At 1 year 72.2% had an SE of ± 0.50 D and 87.0% had an SE of ± 1.00 D	**Distance:** At 1 year, mean monocular UDVA: 0.10 ± 0.11 logMAR CDVA: mean pre-operative: 0.03 ± 0.24 logMAR Mean post-operative: 0.02 ± 0.06 logMAR	**Near:** At 1 year, mean UNVA: 0.18 ± 0.12 logMAR CNVA: mean pre-operative: 0.22 ± 0.09 logMAR Mean post-operative: 0.13 ± 0.08 logMAR		One post-traumatic decentration No significant change in IOP or endothelial cell count	Scheimpflug imaging: Mean distance between the sulcus multifocal IOL and the monofocal IOL was 517 ± 141 µm
**XtraFocus IOL**								
Ho et al. (2022)[69] Retrospective case series	11 eyes, 11 patients 54 years (27 – 81) Mean 11.6 months (4.37 – 18.57)	Mean SE changed significantly (p < 0.05)		**Distance:** Mean UDVA improved significantly (p < 0.05)			Persistent glare and floaters in two patients, both requiring explantation	
Trindade et al. (2017)[70] Prospective case series	21 patients n/a			**Distance:** Statistically significant improvement in UDVA and CDVA Median CDVA improved from 20/200 to 20/50 at one month			No major complications reported	One case of decentration
Trindade et al. (2021)[71] Retrospective consecutive case series	32 eyes, 16 patients 6.9 years 27 months (5 months to 5.5 years)			**Distance:** Mean pre-operative UDVA: 1.09 ± 0.21 logMAR Mean post-operative: 0.34 ± 0.09 logMAR (p < 0.001) at one year	**Near:** No significant improvement		One case of significant inflammation PCO in two eyes	Three cases of decentration requiring repositioning

### The Sulcoflex range (Rayner, Worthing, UK)

**Fig. 1:**
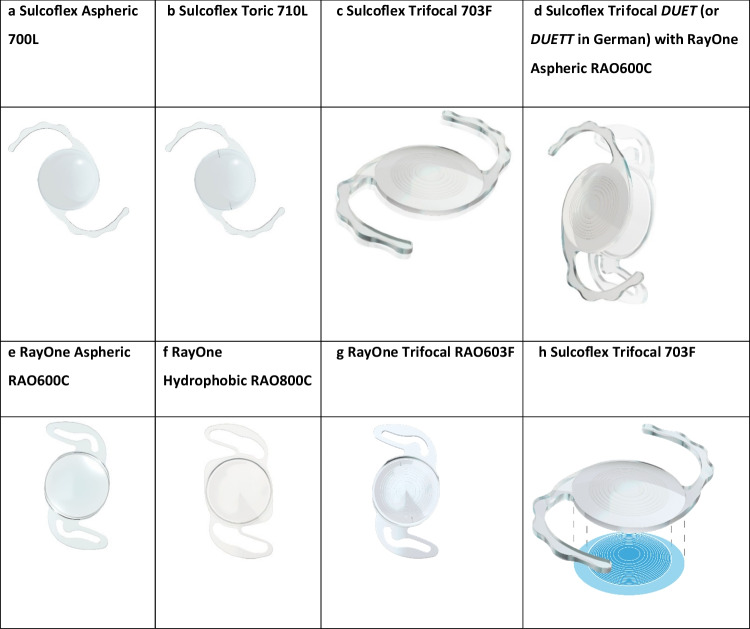
The range of Rayner sulcus-fixated supplementary IOLs

The Sulcoflex range of supplementary IOLs, see Figure 1 (a to h), has been designed to overcome the disadvantages that arise when conventional capsular bag IOLs are placed in the ciliary sulcus.[16] They are available as monofocal, monofocal-toric and trifocal supplementary IOLs. The bifocal refractive multifocal and multifocal-toric models have been discontinued in most markets.

The one-piece hydrophilic design withstands the stress of implantation through a small incision, minimising haptic damage.[56] It has been reported that three-piece IOLs are more likely to lose their “memory” after implantation, causing a shift of the IOL optic. Although these results were obtained from IOLs placed in the capsular bag,[72, 73] laboratory studies confirm that one-piece PMMA lenses exhibit better loop memory compared to three-piece.[74, 75]

The posterior concave design of the Sulcoflex is intended to minimise contact between the optical zones of the two IOLs. A study using Scheimpflug photography to measure the distance between a primary and the secondary Sulcoflex IOL demonstrated that there was a good distance which was maintained over the course of the study between the two IOLs which will significantly reduce the risk of ILO.[56]

In the same study, ultrasound biomicrography (UBM) was used to assess the distance between the Sulcoflex IOL and the iris-pupil edge. Pigment dispersion can occur when the optic margin or haptics chafe the iris pigment epithelium. The UBM images confirmed that the ten-degree angulation of the Sulcoflex keeps the optic at a safe distance from the iris,[56] and this is confirmed by clinical experience. The undulating design of the haptics may also contribute to the stability of the IOL, optimising centration and rotational stability.[56]

#### Optical quality

Łabuz et al (2021) used ray-tracing simulations to assess the effect of the position of an aberration-neutral Sulcoflex IOL on visual performance.[76] The Sulcoflex had a nominal power of 1 to 10 D and was positioned with a capsular IOL of 20 D. The optical performance was tested for misalignment through the area under the modular transfer function (MTFa). There was good tolerance to a 1.0 mm decentration with a loss of MTFa of only 2% for the 10 D Sulcoflex. An extreme 10° tilt would result in a 4% reduction of the MTFa for a 10 D sulcus-fixated IOL, with the impact of off-axis position becoming less noticeable in eye models requiring lower-power supplementary lenses. When misaligned, low-power sulcus-fixated IOLs might retain optical quality more efficiently than a high-power capsular-bag lens, but an extreme tilt has a more detrimental effect on performance than a 1.0 mm decentration.

In an optical bench study, Łabuz et al (2020) compared the MTF and US Air Force (USAF) target images of an aberration-neutral Sulcoflex Trifocal (703F) implanted with an aberration-neutral RayOne Aspheric RAO600C monofocal IOL to that of a capsular bag-fixed aberration-neutral RayOne Trifocal IOL (RAO603F, all from Rayner Intraocular Lenses Ltd).[77] Equivalent results were found for both approaches. The presence of two IOLs rather than one led to a minimal light loss of 1.3% through reflection at additional interfaces, material absorption and light scattering.

#### Stability and clearance

McIntyre et al (2012), using high frequency ultrasound assessment in cadaver eyes already implanted with an IOL in the capsular bag, demonstrated that the Sulcoflex had good centration and minimal or no tilt. The Sulcoflex IOL could be injected and positioned in the ciliary sulcus, giving a clearance between the two IOLs of 232 to 779 µm, depending on the thickness of the primary IOL. The results showed that the design of the IOL minimised the possibility of interaction with the primary IOL and uveal tissues.[59]

#### Centration

Prager et al (2017) confirmed the stability of the Sulcoflex in 48 eyes of 43 patients.[61] After a mean follow-up of 25 months the mean decentration of the Sulcoflex IOL was 0.23 ± 0.02 mm. The mean decentration of the capsular bag IOLs was significantly greater at 0.29 ± 0.02 mm relative to the limbus.

#### Sulcoflex Aspheric 700L (formerly 653L) – aberration neutral monofocal optic

Kahraman and Amon (2010) implanted the Sulcoflex 700L prospectively in 12 eyes of 10 patients requiring correction of residual refractive error.[56] Correction of refractive error was safe and predictable with an improvement in uncorrected distance VA (UDVA) in all cases. There were no signs of pigment dispersion, iris bulging, foreign body giant cell formation, or ILO. There was one case of minor decentration thought to be due possibly to the eye having a large ciliary diameter and weak zonular support.

In another small series, 15 eyes with relatively high residual refractive errors were implanted with the Sulcoflex 700L (13) and the Sulcoflex 653T, now known as the 710T (2).[58] After a mean follow up of 14 months, there was good visual acuity in all patients and predictable refraction. There was no decentration, rotation or tilt in the aspheric lenses.

Venter et al (2014) report a larger study of 80 eyes that had become ametropic following initial multifocal IOL implantation with the Lentis Mplus MF30.[62] Patients with a refractive cylinder of less than 1.0 D were included, and a Sulcoflex Aspheric supplementary IOL ranging from -2.50 to +4.5 D was implanted. At one year, both sphere and cylinder had reduced significantly with 94% of eyes within ±0.50 D of emmetropia, and statistically significant improvements in UDVA and corrected distance visual acuity (CDVA). Implantation of the supplementary IOL did not affect reading performance of the multifocal IOL.

**Key points**: a monofocal aspheric supplementary IOL in the sulcus can be used to correct a residual refractive error after the implantation of a multifocal IOL in the bag. The Sulcoflex Aspheric 700L supplementary IOL is stable in the eye with minimal tilt, decentration or rotation. This IOL provides good visual acuity and predictable refraction. Studies have found a low level of complications, particularly relating to ILO and iris touch.

#### Sulcoflex Multifocal 653F (discontinued in most markets)

Khan and Muhtaseb (2011) successfully implanted four bifocal Sulcoflex Multifocal IOLs and one toric, in five eyes of four patients requiring improvement of their original post-operative vision. All patients had significantly improved UDVA and good uncorrected near VA (UNVA).[43]

Schrecker et al (2014) implanted the bifocal refractive Sulcoflex Multifocal 653F IOL in 35 eyes, and the diffractive MS 714 PB Diff in 33 eyes following implantation of an acrylic IOL in the bag.[51] The IOLs were implanted in a single procedure. At 3 months there was no difference between groups in UDVA or CDVA and both performed well in terms of far, intermediate and near visual acuity. Sixteen out of 19 patients were satisfied or very satisfied with their vision, at all distances and in all lighting conditions. There were more photic phenomena reports in the Sulcoflex patient group but these were mostly rated as mild to moderate. The unfolding process for the Sulcoflex was smoother and more controllable.

The Sulcoflex Multifocal was discontinued in most markets following the launch of the Sulcoflex Trifocal 703F in 2018.

**Key points**: implantation of the bifocal refractive Sulcoflex Multifocal 653F IOL in the sulcus improves intermediate and near visual acuity.

#### Sulcoflex Toric 710T (formerly 653T) – aberration neutral monofocal toric optic

Falzon and Stewart (2012) report that the Sulcoflex Toric IOL is predictable in adjusting post-operative refractive results and reducing spectacle dependence for distance following surgery.[16] In their retrospective case review of 15 eyes, mostly implanted with the Sulcoflex Toric IOL, all patients were within 1.00 D of attempted correction, and 93% within 0.50 D. Astigmatic error was significantly reduced and there were no significant complications.

In a study of ten eyes, Ferreira et al (2015) implanted the Sulcoflex Toric IOL to correct residual astigmatism.[31] Patients had received an IOL in the capsular bag at least 6 months previously, and the refractive error was stable. Good visual results were obtained in all patients: refractive error was significantly reduced and visual acuity improved. There was a statistically significant reduction in ocular aberrometry values and improved photopic contrast sensitivity. This study also provides evidence for the stability of this supplementary sulcus-fixated toric IOL. At 6 months, mean axis misalignment was 3.0 ± 2.45°. As one degree of rotation can lead to a loss of up to 3.3% of cylinder power,[78] this is considered an excellent result by the authors. In difficult cases, where the IOL persists in rotating, as in, for example, cases of irregular sulcus anatomy, the Sulcoflex Toric can be rotated or fixed with transscleral sutures.[79]

McLintock (2019) carried out a retrospective chart review of the Sulcoflex Toric implanted in 51 eyes, including 19 with a history of corneal grafts, with at least 3 months follow-up.[60] Mean UDVA improved significantly but more so in eyes without corneal grafts. Most patients achieved SE within ±0.5 D of target and cylinder. Sixty-two percent required re-positioning, following this, mean rotation was 6.17°.

**Key points**: residual astigmatism can easily be corrected with the supplementary Sulcoflex Toric IOL. Studies demonstrated good stability, leading to minimal misalignment and loss of cylinder power. The intervention is particularly useful for patients not suitable for corneal refractive surgery.

#### Sulcoflex Trifocal 703F – aberration neutral trifocal optic

Kahraman et al (2021), evaluated 40 eyes of 20 patients implanted with a monofocal IOL in the capsular bag and a supplementary diffractive Sulcoflex Trifocal IOL (703F).[57] All patients required an improvement in their visual acuity and had a desire for spectacle independence. At 6 months, visual acuity was good at all distances and all patients reported full spectacle independence. The IOL provided good levels of functional visual acuity over a wide range of defocus values (-3.00 to +0.50 D). Photic phenomena occurred in some patients but were not disturbing. Mean patient satisfaction was 9.21/10. No adverse events were reported for any patients and there was no rotation or tilt. All implanted supplementary IOLs had a base power of 0.00 D, near addition of +3.50 D and intermediate addition of +1.75 D.

In patients scheduled for cataract surgery or refractive lens exchange, Baur et al (2022) carried out combined implantation of the Sulcoflex Trifocal 703F IOL in 25 patients (48 eyes) with implantation of various IOLs in the capsular bag to achieve reversible trifocality.[45] At 3 months, they reported excellent results for far, intermediate and near vision which are comparable to results reported for capsular bag-fixated trifocal lenses. Monocular defocus curve testing found a visual acuity of 0.2 logMAR or better from +0.75 to −3.5 D. There was one case of decentration which required suturing. All patients who completed the assessment reported halos and more than two-thirds of patients reported glare.

In a case report concerning an 18 year old patient with bilateral hereditary hyperferritinaemia-cataract syndrome, a monofocal IOL was implanted in the capsular bag to achieve emmetropia, and a supplementary trifocal IOL in the ciliary sulcus.[80] The supplementary trifocal IOL can easily be removed at a later stage if necessary. A similar procedure was carried out on a young cataract patient with bilateral central posterior shell opacity.[81]

**Key points**: The Sulcoflex Trifocal IOL was stable in the eye and provided good levels of functional visual acuity over a range of distances. As is often the case with trifocal IOLs, photic phenomena were experienced by many patients but were generally not disturbing.

### The AddOn range (1stQ GmbH, Mannheim, Germany)

**Fig. 2:**
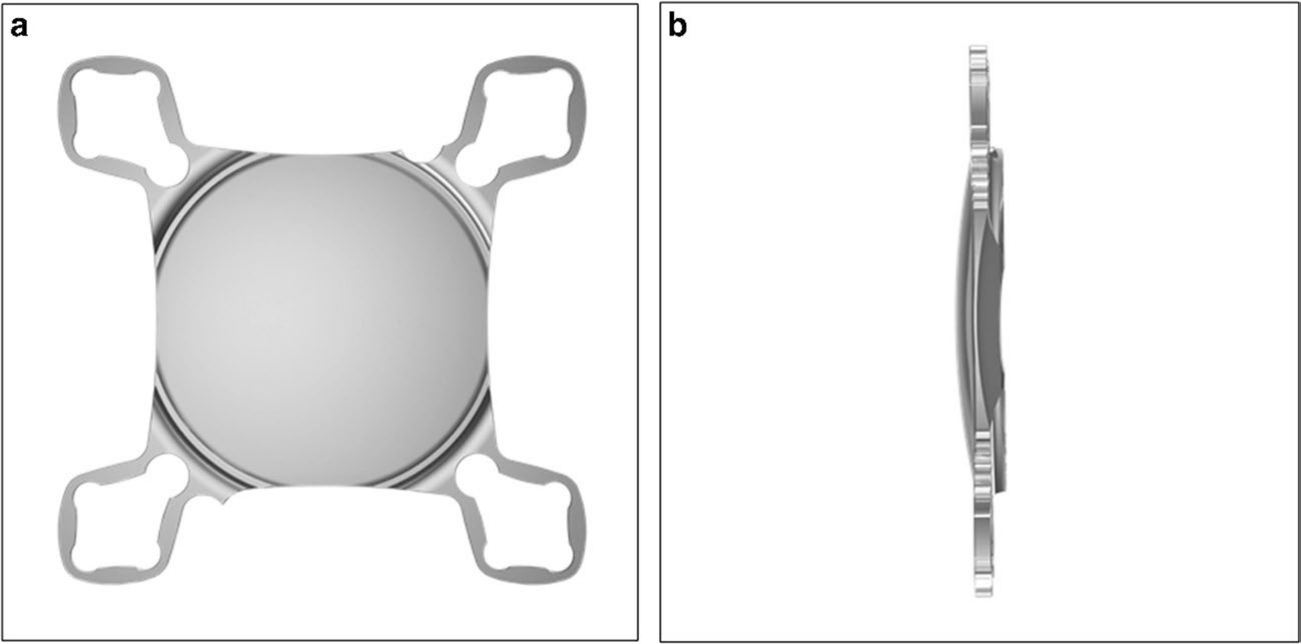
1stQ AddOn IOL

The AddOn range of IOLs are single piece devices composed of hydrophilic acrylic. They have four square-shaped haptics and a large optic designed to avoid pupillary capture along with round edges to minimise iris chafing, see Figure 2.[67]

They are available as monofocal-spherical, monofocal-toric, multifocal (bifocal diffractive), multifocal-toric and trifocal, with different power additions including low add, or extended depth of focus versions. The term “AddOn” has sometimes been used loosely in the published literature to mean any supplementary IOL, so caution is needed in interpreting studies to ensure that the IOL is part of the 1stQ range.

#### Stability

Reiter et al (2017) implanted the 1stQ AddOn supplementary IOL into the ciliary sulcus of 12 cadaver eyes that had previously been implanted with a range of capsular bag IOLs. Assessment with anterior segment optical coherence tomography (AS-OCT) found the AddOn to be well centred in all eyes. Four cases of tilt were seen: three with mild tilt due to pre-existing zonular dehiscence, and one due to a localized area of Soemmering’s ring formation. Mean distance between the two IOLs was 0.68 mm (0.34 to 1.24). Overall the IOL demonstrated proper fixation and centration.[67]

#### AddOn Spherical / Toric IOL

Gundersen et al (2017) carried out a chart review of 46 eyes implanted with the supplementary spherical or toric AddOn IOL and a variety of primary IOLs. Following implantation of the supplementary IOL, there was a statistically significant improvement in UCVA of about 2 lines, with no change in BCVA and a significant reduction in the absolute magnitude of the residual spherical equivalent refractive error. In the ten cases with a toric secondary IOL, there was a statistically significant reduction in astigmatism. The IOL provides a viable surgical option to correct residual refractive error after primary IOL implantation and performance was unrelated to the identity of the primary IOL.[64]

Gundersen et al (2020) evaluated eighteen eyes with secondary implantation of the AddOn toric supplementary IOL at least one month after surgery. Mean residual refractive astigmatism was significantly reduced. All patients had very good visual acuity. There was little change in orientation since implantation with mean absolute lens rotation of ≤5°: 89% of eyes had a lens rotation of ≤10° and only two eyes had a lens rotation of more than 10°.[65] The authors concluded that lens rotation was minimal.

**Key points**: the 1stQ AddOn spherical IOL improves visual acuity and reduces residual SE while the toric model can significantly reduce astigmatism.

#### AddOn Progressive 677MY (Trifocal IOL)

Palomino-Bautista et al (2020) implanted the 1stQ AddOn trifocal supplementary IOL in 18 eyes of 11 pseudophakic patients requiring improved spectacle independence.[42] All patients had previously undergone uncomplicated implantation of monofocal capsular bag IOLs. Of interest is that the mean time after implantation of the primary IOL was 11.4 years. After surgery, 83.3% of eyes had spherical refractions within ±0.5 D of emmetropia and 100% of eyes had spherical equivalent refractions within ±1.0 D of target refraction. Visual acuity and defocus curves confirmed the trifocal nature of the IOL which was superior in intermediate and near ranges compared to a trifocal capsular bag IOL. All patients achieved spectacle independence at all distances. All AddOn IOLs were well positioned in the ciliary sulcus. The authors conclude that the supplementary trifocal AddOn IOL is a safe, efficient and stable solution for achieving spectacle independence in pseudophakic patients.

Albayrak et al (2021) implanted the AddOn trifocal supplementary IOL in a prospective study involving 28 eyes of 18 patients who had been implanted with a primary IOL in the previous year.[63] Mean UNVA improved significantly and results for intermediate VA were good. Twenty-five eyes had a residual SE within 1.0 D of target refraction. Contrast sensitivity was unaffected. All patients had better visual function and quality scores compared to pre-operative values, with the highest improvement being seen in near vision.

Harrisberg et al (2023) carried out a retrospective analysis of patients implanted with a trifocal AddOn (A4DW0M) secondary to implantation of a toric or non-toric monofocal in the bag, comparing the results to those obtained from a single capsular bag multifocal.[66] They concluded that the IOL was effective and safe in correcting distance and near vision and increasing spectacle-independence. There were no significant differences between the groups in any parameter.

Khoramnia et al (2023) performed a laboratory analysis of a trifocal AddOn (A4DW0M) in a polypseudophakic model with a monofocal lens compared to a trifocal IOL (B1EWYN) in the capsular bag.[82] The MTF Strehl ratio was measured, and the USAF-target images were assessed. The polypseudophakic system yielded a better optical quality at far focus, while the standard trifocal provided improved imaging at near distances. The differences arose from different diffractive patterns between the two models; however, visual inspection of the resolution chart did not reveal noticeable image-quality loss confirming the equivalence of the two approaches.

**Key points**: the AddOn Progressive trifocal IOL from 1stQ provides predictable and stable refraction, good visual acuity at all distances and a high rate of spectacle independence.

### Reverso (Cristalens International SAS, Lannion, France)

**Fig. 3:**
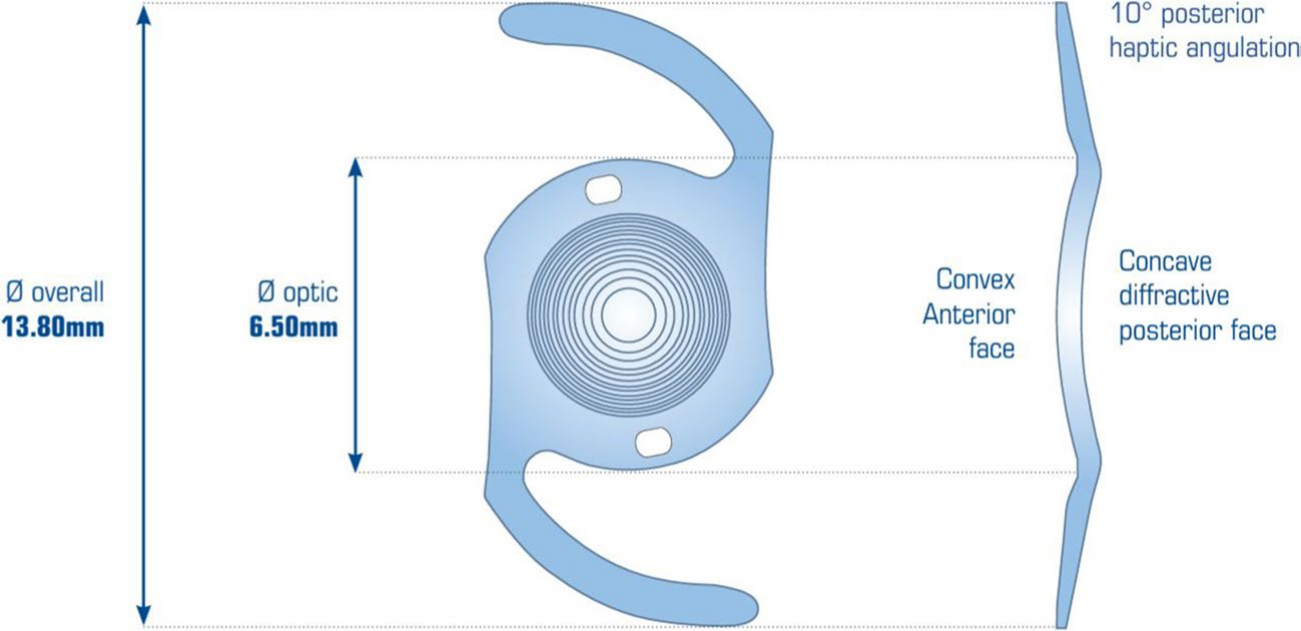
The Cristalens Reverso® IOLs

The Reverso multifocal IOL (see Figure 3) was implanted in a primary procedure along with a capsular bag IOL, into the eyes of 27 patients undergoing cataract surgery, and seeking presbyopia correction.

[68] At one year, there was improvement in uncorrected distance and near vision, a high level of patient satisfaction and a low level of complications.

#### XtraFocus pinhole IOL (Morcher GmbH)

The XtraFocus pinhole IOL offers a different approach to correction of residual refractive errors but tends to be used in eyes with high corneal aberration, irregular astigmatism, or large or irregular pupils.

**Fig. 4:**
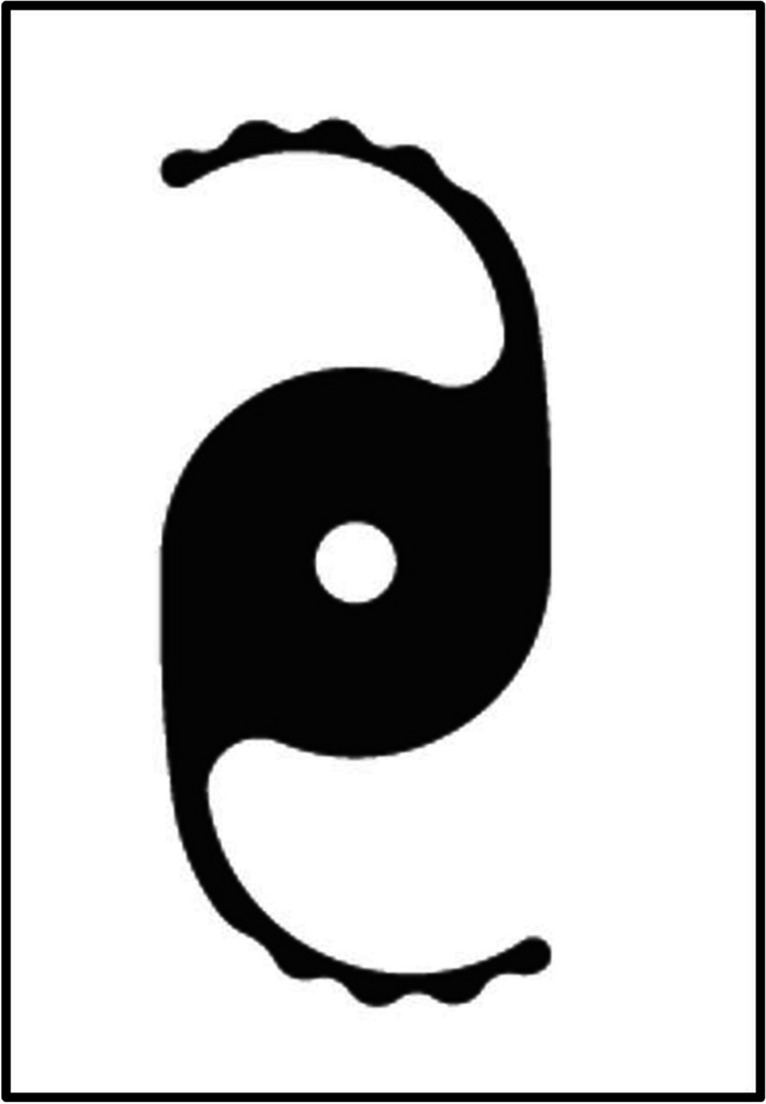
The Morcher XtraFocus pinhole supplementary IOL

The XtraFocus IOL is composed of black hydrophobic acrylic which blocks visible light but is transparent to infrared light to permit retinal examination. The central section is 6 mm in diameter including the occlusive section, with a concave‑convex design and no refractive power, see Figure 4.

In the middle is a central opening of 1.3 mm, and the IOL has an overall diameter of 13.5 mm. The haptics are rounded, polished and undulating and are 250 μm thick to avoid uveal tissue injury and are angulated at 14° to prevent iris chafing and pigment dispersion. The XtraFocus received the CE mark in 2016 and is commercially available in several countries.[54]

#### Applications

In patients with clear central corneas, the Xtrafocus can neutralise high amounts of corneal aberration and extend the depth of field for presbyopia correction. Patients with significant dysphotopsia from multifocal IOLs or severe glare and light sensitivity from irregular or large pupils may also benefit.[54] The device is helpful in pseudophakic patients with irregular astigmatism causing significant visual impairment,[70] and can also be beneficial in patients with large iris defects.[71]

Secondary indications are near or intermediate vision enhancement in pseudophakes with monofocal IOLs, and dysphotopsia reduction in eyes with multifocal IOLs.[69]

#### Clinical studies

There are a limited number of clinical reports for the XtraFocus. Trindade et al (2017) implanted the XtraFocus to correct irregular corneal astigmatism in 21 patients with significant visual impairment due to keratoconus, radial keratotomy or penetrating keratoplasty, traumatic corneal laceration, post‑LASIK ectasia, and eccentric excimer laser ablation. There was marked improvement in visual function and high patient satisfaction following surgery.[70]

Ho et al (2022) implanted the XtraFocus in 11 patients with irregular corneal astigmatism, or with iris trauma. The device was implanted at the same time as a capsular bag IOL, but all XtraFocus IOLs were implanted in the sulcus. The procedure was effective at improving vision or reducing glare but two patients had to have the device explanted because of severe glare.[69]

The XtraFocus was implanted in 32 eyes of 16 patients with irregular corneal astigmatism and BCVA worse than 20/50 in both eyes. Patients had undergone surgery either for clear lens exchange or due to cataract. The IOL was found to be safe, and effective, bringing about significant improvement in visual acuity.[71] Note: in just over half of the cases, both primary and supplementary IOLs were implanted in the capsular bag. The results for the two procedures are not reported separately.

A publication in 2020 reported a retrospective study of 60 eyes implanted with the device to treat irregular corneal astigmatism.[83] However, in this study, the device was implanted in the capsular bag alongside a variety of other IOLs so will not be considered here. The authors state that this IOL is less stable in the sulcus compared to placement in the capsular bag.

To summarise, IOLs designed for use in the ciliary sulcus have been specifically designed to overcome the disadvantages that arise when conventional capsular bag IOLs are used in this way.

Important features of the Sulcoflex range are a posterior concave design to minimise contact between the optical zones of the two IOLs and sufficient angulation of the haptics to keep the optic at a safe distance from the iris. The AddOn range employs a different design with four square-shaped haptics and a large optic intended to avoid pupillary capture. Rounded edges on the haptics are a common feature of both IOLs to minimise iris chafing. The XtraFocus supplementary IOL is a pinhole device intended for the correction of irregular residual astigmatism and other more specialist applications.

Both main IOL ranges have demonstrated proper fixation and centration, and one study confirmed better centration of the Sulcoflex platform in the ciliary sulcus compared to IOLs implanted in the capsular bag. However, there is insufficient published data on the Reverso IOL to reach a conclusion on stability, and one study on the XtraFocus suggested it was less stable in the sulcus.

The Sulcoflex monofocal aspheric supplementary IOL can be used to correct residual refractive errors after implantation of other IOLs in the bag, providing good visual acuity and predictable refraction. The bifocal refractive Sulcoflex Multifocal 653F IOL improves intermediate and near visual acuity, while the Sulcoflex Trifocal IOL provides good levels of functional visual acuity over a range of distances. Photic phenomena are experienced by many patients but in both cases the procedure is easily reversible if the patient cannot adjust. Residual astigmatism can easily be corrected with the supplementary Sulcoflex Toric IOL which has demonstrated good stability, leading to minimal misalignment and loss of cylinder power.

The 1stQ AddOn spherical IOL improves visual acuity and reduces residual SE while the toric model significantly reduces astigmatism. The AddOn Progressive trifocal IOL provides predictable and stable refraction, good visual acuity at all distances and a high rate of spectacle independence.

## Power calculations for supplementary IOLs.

Unlike implantation into the capsular bag, it is a very simple process to find the appropriate power for a supplementary IOL, depending on the refractive target. If the target is emmetropia, all that is required is the patient’s current and stable subjective refraction (spectacle prescription). There is usually no need for biometry data or other complex measurements. It is also not necessary to know the power of any IOL in the capsular bag: the optical power of the supplementary IOL is simply calculated based on the existing subjective refraction of the optical system.

In pseudophakic patients, supplementary sulcus fixated IOLs are usually required in relatively low powers, therefore, deviations from the optimal lens positioning should be of limited clinical relevance and importance.[45]

Each manufacturer has provided online calculators, specific to each IOL which can be found on the individual manufacturer’s website. The following links were correct at the time of going to press:

• Sulcoflex, Raytrace (Rayner): Raytrace—Rayner (https://rayner.com/en/raytrace/)
• AddOn, 1stQ / Medicontur: https://www.1stq.de/en/iol-calculator
• Reverso, Cristalens: Cristalens calculateur – Cristalens (https://cristalens.fr/calculateur/page-calculator)

Alternatively, Amon et al (2023) recommended a simple calculation method:

• R-vergence formula: spherical equivalent (SE) of ametropia, K-values, ACD
• Post-operative ametropia within ± 7.0 D
  o Hyperopia: spherical equivalent (SE) × 1.5
  o Myopia: spherical equivalent (SE) × 1.2

Although emmetropia is the most common aim, targeting myopia with the capsular bag IOL can be useful when aiming to correct presbyopia with a multifocal or trifocal supplementary IOL.[84] In Myopic Multifocal Duet Implantation (MMDI) the target refraction of the primary capsular bag–implanted IOL can be set at approximately −2.5 D to optimise the refraction for near distance should the supplementary IOL be removed at a later date.[85]

The MMDI approach was used for two of 25 patients in a recent study.[45] The RayTrace Premium IOL calculator was used for the supplementary sulcus-fixated Sulcoflex IOLs, targeting for emmetropia to compensate for the myopic target refraction of the primary IOL. The target refraction of the capsular bag–fixated IOL was emmetropia in the 23 other cases and a supplementary sulcus-fixated IOL with plano power was therefore selected.

## Conclusions

The term “Piggyback IOL” should ideally not be used any more to describe the use of two IOLs in the eye, as it originated as a description for procedures that are associated with many potentially serious complications: the two-in-the-bag approach, and the implantation into the ciliary sulcus of an IOL designed for the capsular bag.

“Supplementary IOL implantation” is the preferred term for the addition of a second IOL which has been specifically designed for implantation in the ciliary sulcus, to an eye that has another IOL in the capsular bag. The two implantations may be carried out sequentially or in the same procedure. Other terms in use are DUET procedures or secondary enhancement. Critical design features include a concave posterior surface of the IOL to maintain distance between the two optics, and angulated haptics that minimise contact with the iris.

Implantation of an appropriately designed supplementary IOL is safe and effective as a means of correcting residual refractive error. The reversibility of the procedure can also offer the surgeon many other options for patients, such as reversible trifocality, refractive correction in patients with temporary tamponades or with ongoing ocular pathologies, and in developing eyes.
